# Microbiome and Heart Failure: A Comprehensive Review of Gut Health and Microbiota-Derived Metabolites in Heart Failure Progression

**DOI:** 10.3390/medsci13040302

**Published:** 2025-12-04

**Authors:** Chukwudi Kingsley Orjichukwu, Rita Ogochukwu Orjichukwu, Peter Kanayochukwu Akpunonu, Paul Chikwado Ugwu, Somtochukwu Godfrey Nnabuife

**Affiliations:** 1 Well Health Manitoba Clinic, 790 Sherbrook Street, Winnipeg MB R3A 1M4, Canada; 2 Medway Maritime Hospital, Gillingham ME7 5NY, UK; 3 North London NHS Foundation Trust, IT Department, 3rd Floor, West Wing, St. Pancras Hospital, 4 St. Pancras Way, London NW1 0PE, UK; 4 King George Hospital, Barley Lane, Ilford IG3 8YB, UK; 5 Cranfield University, Central Ave., Cranfield, Bedford MK43 0AL, UK; 6 University of Wolverhampton, Wulfruna Street, Wolverhampton, West Midlands WV1 1LY, UK

**Keywords:** gut microbiome, heart failure, microbiota-derived metabolites, trimethylamine N-oxide (TMAO), short-chain fatty acids (SCFAs)

## Abstract

A multifaceted clinical disease, heart failure (HF) is typified by decreased cardiac function and systemic symptoms caused by anatomical or functional abnormalities in the heart. Although traditional studies have concentrated on hemodynamic and neurohormonal processes, new data highlight the vital role that the gut microbiota and its byproducts play in the pathogenesis of HF. An imbalance in the microbial structure known as gut dysbiosis is common in HF patients and is linked to increased gut permeability, systemic inflammation, and changed bioactive metabolite synthesis. Prominent metabolites generated by the microbiota, including phenylacetylglutamine, short-chain fatty acids (SCFAs), secondary bile acids, and trimethylamine N-oxide (TMAO), have a major impact on endothelial function, cardiac remodeling, and inflammation. Together with gut-derived lipopolysaccharides, these metabolites interact with host systems to exacerbate the course of HF. Further impacting HF outcomes are comorbidities such as diabetes, obesity, and chronic renal disease, which intensify gut dysbiosis. The importance of metabolites originating from the microbiota in the progression of HF is highlighted in this review, which summarizes recent findings regarding the gut-heart axis. Additionally, it investigates how dietary changes, probiotics, prebiotics, and multi-omics techniques can all be used to improve the management of HF. This thorough analysis emphasizes the necessity of integrative therapy approaches and longitudinal research to better address the complex link between HF and the gut microbiota.

## 1. Introduction

Heart failure (HF) is a clinical syndrome characterised by impaired cardiac function and systemic symptoms resulting from structural or functional abnormalities [1]. HF affects well over 64 million people worldwide and continues to increase in prevalence, leading to significant reductions in quality of life and substantial healthcare expenditure [2]. In the United States alone, HF-related costs exceeded $30 billion in 2012 and are projected to nearly double by 2030 [3,4,5,6]. HF outcomes differ markedly worldwide, with low-income areas demonstrating diminished health-related quality of life and elevated mortality rates in contrast to Western Europe. The clinical and economic burden of HF suggests that there is an improved understanding of contributing mechanisms and innovative therapeutic approaches, particularly regarding emerging evidence linking gut dysbiosis and microbial metabolites to the progression and prognosis of HF [7,8,9,10,11].

The gut microbiota represents a dynamic and integral component of the human body, acquired at birth, that performs essential functions within its metabolic, structural, neurological and immunological frameworks. It also exerts significant influence on both physical and mental health. Studies [12,13,14,15] have demonstrated that the gut microbiota can be involved in the pathogenesis and progression of cardiovascular diseases (CVDs), including HF [16]. Furthermore, both the gut microbiota and its associated metabolites have been implicated in HF [17]. Various CVDs such as hypertensive heart disease, atherosclerosis, myocardial infarction, heart failure and arrhythmia have been linked to altered intestinal flora [18]. Moreover, gut microbial fermentation metabolites play a role in the development, prevention, treatment and prognosis of CVDs; these metabolites include trimethylamine N-oxide (TMAO), short-chain fatty acids (SCFAs), secondary bile acids (BAs) and gases such as hydrogen sulfide (H_2_S), carbon dioxide (CO_2_) and nitric oxide (NO) [19]. The relationship between gut microbiota and the biological processes that influence CVD risk is complex; however, it is crucial to understand these interactions because they could inform potential therapeutic strategies [20].

However, there is currently limited research on the direct effects of gut microbiota-associated metabolites in HF. Therefore, a comprehensive review is needed to better understand their mechanistic roles and potential therapeutic relevance. A clearer understanding of these interactions may support the development of novel microbiome-targeted strategies for HF management. But we must proceed with caution, as the complexities of these interactions are not yet fully elucidated. This paper presents a thorough examination of the gut microbiota’s role and its metabolites in the progression of HF. The investigation synthesizes contemporary evidence that correlates gut dysbiosis, microbial-derived metabolites, and systemic inflammation with HF pathophysiology. It underscores the influence of metabolites such as TMAO, SCFAs, and secondary bile acids on cardiac remodeling and immune responses, thus emphasizing their potential as biomarkers and therapeutic targets. Furthermore, the paper delves into the interaction between gut health and comorbidities, including diabetes and obesity, which exacerbate HF outcomes. By integrating insights derived from dietary, probiotic, and multi-omics approaches, it suggests innovative strategies for modulating gut microbiota to manage HF, highlighting the necessity for multidisciplinary research and longitudinal clinical trials.

The paper is systematically organized as follows: Section 1 provides an overview of HF, its global burden, and its intricate relationship with gut microbiota, thereby stressing the imperative for a comprehensive review. Section 2 delves into the multifaceted functions of the microbiome and its correlative impact on host health. Section 3 elucidates the various metabolites, such as TMAO, SCFAs, bile acids, and phenylacetylglutamine, articulating their significant roles in the progression and prognosis of HF. Section 4 summarizes a plethora of studies that establish a connection between gut microbiota and HF; however, Section 5 discusses a range of interventions, including probiotics, prebiotics, dietary modifications, fecal microbiota transplantation, and renal denervation. Although these interventions are promising, they highlight the necessity for future research priorities, which should encompass personalized approaches and multi-omics methodologies. Section 6 concludes by underscoring the critical importance of integrating gut microbiota research into the management of HF as well as emphasizing the imperative for multidisciplinary efforts to enhance our understanding of this complex interplay.

- Gut dysbiosis contributes to HF via systemic inflammation and endotoxemia.
- Microbial metabolites, like SCFAs and TMAO, affect cardiac remodeling and function.
- Comorbidities, like obesity and diabetes, exacerbate gut dysbiosis in HF.
- Dietary and probiotic interventions hold potential for microbiome-targeted HF therapies.

## 2. Methodology

To assess the available literature on gut microbiota, microbial-derived metabolites, and heart failure, a thorough literature search was performed using a structured process. The authors searched arthouses (PubMed, Scopus, and Web of Science), as well as Google Scholar, for literature published from 2010 to 2024. The following keywords could have been used in combination with concentrators: “gut microbiota” AND “heart failure”, “gut dysbiosis”, “microbiome metabolites”, “TMAO”, “SCFAs”, “bile acids”, and “cardiac remodelling”.

Both preclinical and clinical studies were included: observational studies, randomised controlled trials, meta-analyses, and mechanistic reviews. Exclusion criteria included any case report, conference abstract without a full text, or study that did not examine cardiovascular implications of the gut microbiome. Inclusion criteria consisted of peer-reviewed articles written in English that studied gut microbiota composition, metabolites, or treatment strategies related to heart failure.

The first search returned over 1200 articles, from which we chose 158 based on relevance and methodological quality after screening titles and abstracts, and 94 after full-text review. We also hand-searched the reference lists of key articles for additional eligible studies. Although this review did not entail a complete systematic review protocol, a structured search process was used to maximise coverage of evidence without bias.

## 3. Science of the Gut Microbiome: Present Knowledge

**Figure 1:**
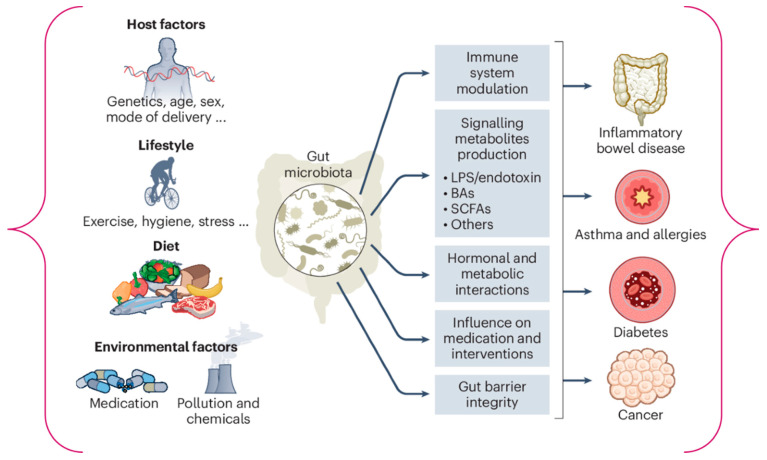
Lifetime Gut Microbiome and Key Environmental and Host Determinants [23].

The gut microbiome functions at a pivotal intersection between the host organism and its surrounding environment, effectively modulating host physiology in ways that can be astonishingly individualized [21]. The degree of similarity in gut microbiome composition among individuals irrespective of familial connections is significantly low. As depicted in Figure 1, numerous factors assume crucial roles in sculpting the microbiome and nurturing this diversity; however, in contrast to host genetics, environmental determinants such as dietary practices and pharmacological interventions appear to exert a much more substantial influence [22]. Furthermore, Figure 1 elucidates the predictable transformations in the microbiome across the human lifespan, underscoring the fragile balance between the microbiome’s resilience and its malleability when faced with perturbations. This intricate interplay is essential for understanding the complexities of microbiome dynamics in human health.

The composition of the gut microbiome is predominantly shaped by environmental influences (such as diet, medications and lifestyle choices); host genetics contribute to this shaping, albeit to a lesser extent. However, although alterations in these environmental factors can precipitate microbial shifts, the microbial community generally exhibits a remarkable degree of stability and resilience. This stability is only disrupted by substantial perturbations, particularly after the toddler years when the adult microbiome becomes “established” and remains relatively stable until advanced age.

Table 1 offers a comprehensive overview of the most common methodologies, emphasizing their strengths and weaknesses. These approaches can be employed in research involving either humans or animals. By promoting a reductionist framework for hypothesis testing, animal models especially those that use germ-free subjects lacking resident microbiota have greatly enhanced our comprehension of the microbiome-host relationship. However, the transfer of these findings to human applications has faced challenges; in addition to their failure to completely capture the intricacies of human biology and its numerous environmental factors, animal research has shown that some microbiome-host interactions are specific to particular host species. This indicates that the crucial element for effective human translation is the meticulous selection of model systems that authentically replicate the disease characteristics of concern, at both the host and microbiome levels. Relevant instances can be observed in porcine models of metabolic syndrome and HF with preserved ejection fraction, where both host and microbiome physiology, along with their reactions to disturbances, closely resemble those encountered in humans [24].

### 3.1. Gut Microbiome: A Tool for Optimizing Heart Failure Therapy

The interplay between pharmacological agents and gut microbiota constitutes a bidirectional relationship; specifically, medications have the capacity to modify the microbiome [25], whereas microbial entities can influence the pharmacokinetics and pharmacodynamics of various drugs [26]. A precision medicine framework that acknowledges these critical interactions holds the potential to enhance HF therapies, thereby enabling patients to utilize medications that yield individualized benefits while minimizing adverse effects. Numerous widely prescribed cardiovascular agents, such as beta-blockers, RAS inhibitors, digoxin, calcium-channel blockers, statins and antiplatelets, are metabolized by gut microorganisms. For instance, digoxin, an agent indicated for the management of chronic HF and atrial fibrillation, is selectively inactivated by a prevalent gut microbe, Eggerthella lenta; however, this inactivation occurs exclusively via strains possessing a specific gene [27]. The suppression of this gene in murine models led to increased serum levels of digoxin. Furthermore, it was a protein-rich diet that resulted in the downregulation of this gene [27], thereby demonstrating the downstream modulation of pharmacological effects not solely by gut microbiota but also through intricate microbiome-dietary interactions.

Microbiome profiles possess the potential to forecast drug responses and inform therapeutic decisions. A compelling demonstration of this methodology emerges from the realm of oncology. The antitumor efficacy of immune checkpoint inhibitors (ICIs) is influenced by the gut microbiome and its intricate interactions with the immune system. Distinct microbiome profiles have been correlated with varied ICI responses [28]. Certain microbiome signatures have already been effectively utilized in patients afflicted with lung cancer to anticipate future ICI responses [29]. A comparable strategy could be adopted in the context of chronic HF, where microbiome profiles might be harnessed to predict clinical outcomes related to specific HF treatments. This could facilitate the development of more personalized therapeutic regimens. However, the complexities inherent in the microbiome-immune system relationship necessitate careful consideration, because understanding these interactions could lead to significant advancements in patient care.

Microbiome-targeting therapies may indeed become an integral component of future HF treatment strategies. Probiotics defined as foods and dietary supplements harboring live microorganisms, engage with the gut microbiota, thereby beneficially modifying host physiology. Certain probiotics might specifically modulate pathogenic processes that are dysregulated in HF, as evidenced in a rodent model where the administration of Lactobacillus- and Bifidobacterium-containing probiotics resulted in significantly enhanced cardiac function. However, the GutHeart trial, a randomized investigation assessing the efficacy of the probiotic yeast Saccharomyces boulardii in individuals with stable HFrEF, revealed no notable improvement in cardiac function when compared with standard care [30]. Although these findings are disheartening, the negative outcomes of GutHeart should prompt a recalibration of our approach toward evaluating probiotics. This recalibration necessitates the identification of microbiome “probiotypes” that can provide appropriate ecological niches for distinct probiotics, prior to assessing the clinical efficacy of these interventions.

### 3.2. The Gut Microbiota’s Role in Pathology and Physiology

The human gastrointestinal tract functions as a dynamically balanced micro-ecosystem, wherein more than 2000 species and approximately 100 trillion microbes, predominantly bacteria, viruses, and fungi, exist and coevolve with us (this is an intricate symbiotic relationship) [31]. Bacteria constitute the majority of the gut microbe species, with over 90% represented by Bacteroidetes and Firmicutes, subsequently followed by Actinobacteria, Tenericutes, and Proteobacteria [32]. It is noteworthy that the gut microbiota typically establishes itself in the oxygen-devoid, nutrient-abundant ascending colon, which serves as an optimal habitat for survival. Although the gut microbiota begins to colonize and mature from the moment of birth, its composition and functionality can be influenced by various external factors. Through the breakdown of dietary components and the synthesis of vital vitamins like B and K, the gut microbiota supports the host’s growth, metabolism, and other developmental processes. However, the gut microbiota’s production of SCFAs through the fermentation of dietary fiber offers the host protection in a variety of ways, such as influencing the immune system and providing energy for enterocytes [33]. Furthermore, by secreting signals that encourage epithelium renewal and the induction of intestinal vascular remodeling, microorganisms help to maintain intestinal growth and integrity [34]. This interaction emphasizes how important the gut microbiota and its metabolites are to maintaining the host’s health. The change in gut microbiota composition and function, known as gut dysbiosis, is caused by a number of reasons, including antibiotic abuse, intestinal inflammation, cold stimulation, and other variables. But according to a number of studies, gut dysbiosis is connected to a wide range of illnesses, such as human cancer, irritable bowel syndrome, and cardiovascular disease. It has also been connected to the newly discovered coronavirus disease 2019 (COVID-19). This syndrome contributes to the development of cardiovascular illnesses by impairing the integrity of the gut barrier, increasing intestinal inflammation, and increasing the absorption of microbial products and metabolites into the host’s circulation. Mechanical findings into the impact of gut microbiota in cardiovascular disorders span from inflammation, immunology, and vascular function modulation to reactive oxygen species (ROS) regulation and lipid metabolism [35]. Overall, the gut microbiota has a role in disease pathogenesis, including cardiovascular disease.

## 4. Gut Metabolites Associated with HF

The gut microbiota performs an essential role in the intricate degradation of carbohydrates, proteins and, to a somewhat lesser extent, fats alongside various other biomolecules, which includes the fermentation of non-digestible substrates [36]. Certain metabolites produced via this elaborate process have been linked to heart failure [37]; however, the most consequential among them will be explicated in detail in the subsequent sections. Although this association necessitates additional scrutiny, it remains imperative to contemplate the ramifications of these metabolic byproducts. Because their functions can be diverse, comprehending them is of utmost importance.

### 4.1. Short-Chain Fatty Acids: Protective Roles and Mechanisms in HF

SCFAs, including acetate, propionate, and butyrate, are largely found in the colon; nonetheless, they have the capacity to circulate throughout the body, exerting a variety of physiological effects [38,39]. SCFA-producing bacteria, such as *Faecalibacterium prausnitzii*, as well as members of the *Lachnospiraceae* and *Ruminococcaceae* families, were found to be significantly lower in individuals with HF. These bacterial communities are required for the formation of butyrate; hence their presence suggests possible cardio protective benefits [40]. In the context of various CVDs, a lower prevalence of butyrate-producing species has been associated with an increased risk of developing atherosclerosis. Though preclinical studies suggest that increasing dietary SCFA intake may improve heart function, the precise processes and effects of SCFAs for HF remain unknown, and further study is needed to clarify these complicated interactions [41].

SCFAs are known for their immunomodulatory capabilities, which affect cardiac structure and function, primarily via activating anti-inflammatory regulatory T cell systems [42]. Furthermore, SCFAs may modulate blood pressure, possibly leading to the development of HF; however, more research is needed to substantiate this link. Notably, SCFAs, particularly butyrate, play an important role in gut barrier integrity by promoting intestinal epithelial cell differentiation, repairing damaged mucosa, increasing tight junction protein expression, stimulating mucus production, and reducing inflammation induced by circulating exogenous substances. These consequences may also include responses to external stresses, which could reverse negative alterations [43]. Butyrate is thought to activate hypoxia-inducible factor (HIF) in the colon, maintaining the effectiveness of the gut barrier in the physiologically hypoxic environment of the area. Reduced SCFA levels may be a contributing factor to the progression of HF, according to findings of increased gut permeability in HF patients [40]. Despite these results, there is still a substantial lack of research on the function of SCFAs in decompensated heart failure, with no studies, as far as we are aware, examining their effects on patient outcomes in this particular patient group.

From the mechanistic stand-point, decreased availability of SCFAs has been demonstrated to further the progression of HF through multiple mechanisms. Lower levels of butyrate and acetate reduce intestinal barrier integrity, thus increasing intestinal permeability and endotoxin acting as lipopolysaccharides (LPS) to enter the circulation and elicit a systemic inflammatory response and myocardial damage [42]. Additionally, SCFA deficiency decreases G-protein-coupled receptors (GPR41 and GPR43) activation pathways affecting immune modulation and increases pro-inflammatory cytokines including TNF-α and IL-6, both of which have strong links to adverse cardiac remodelling and systolic dysfunction [40]. Furthermore, low levels of SCFA decrease energy availability to colonocytes and the failing myocardium contributing to mitochondrial damage, oxidative stress, and contractility dysfunction [44]. Overall, these mechanisms collectively show how low levels of SCFAs may directly promote pathophysiological mechanisms that can worsen outcomes in HF.

**Figure 2:**
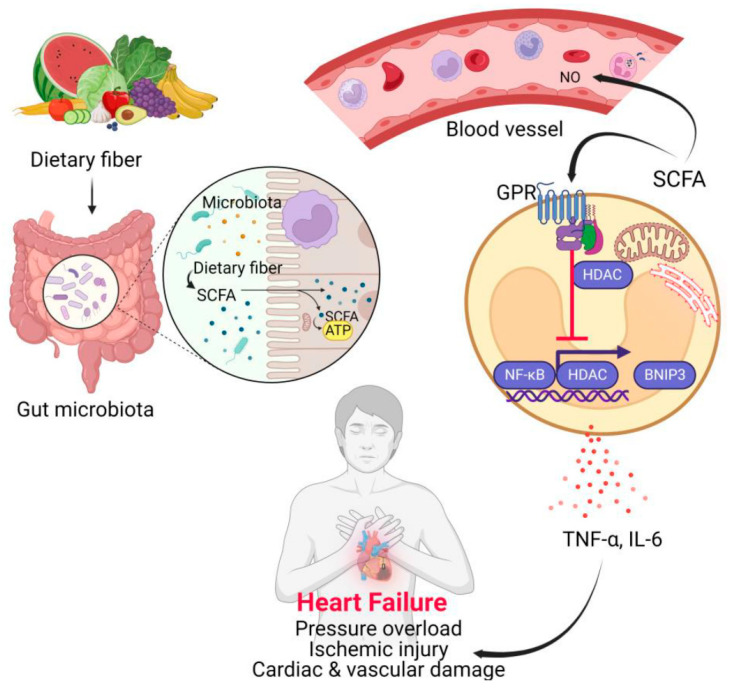
The role of SCFAs in heart failure [45].

The role of SCFAs in the context of heart failure presents an intriguing area of inquiry (see Figure 2). These compounds are synthesized from dietary fiber through the fermentation processes conducted by gut microbiota. SCFAs serve a vital function by providing energy to enterocytes and various innate immune cells. However, their influence extends beyond mere energy provision; they engage with endothelial cells and immune cells through signaling mechanisms mediated by G-protein-coupled receptors (GPRs). This interaction leads to the repression of nuclear factor kappa-light-chain-enhancer of activated B cells (NF-κB), which occurs in conjunction with histone deacetylases (HDACs). Consequently, SCFAs inhibit the expression of Bcl-2 interacting protein 3 (BNIP3), thereby mitigating the production of pro-inflammatory cytokines. These cytokines are known contributors to cardiac and vascular damage, which can result in pressure overload, ischemic injury, and ultimately, heart failure. In this complex interplay, components such as tumor necrosis factor alpha (TNF-α), NO, and adenosine triphosphate (ATP) are also implicated, further underscoring the multifaceted role of SCFAs in cardiovascular health. Although the mechanisms are intricate, the implications of SCFAs in modulating inflammation and cellular responses remain a pivotal aspect of understanding heart failure pathology.

Preclinical evidence suggests that SCFAs may exert cardioprotective effects, including improvements in mitochondrial ATP production and myocardial contractility; however, these findings primarily originate from animal studies and require validation in human HF populations. Peters et al. [46] demonstrated that butyrate supplementation improved cardiac contractility and mitochondrial energetics in experimental heart failure models, whereas Yang et al. [47] and Organ et al. [48] reported that modulation of microbial metabolite pathways may alter HF progression in vivo. While these data offer significant mechanistic insights, they have not yet established a definitive causal relationship in human heart failure; thus, these associations should be regarded with caution. Further, randomised control trials are required to determine whether SCFA supplementation or microbial manipulation can meaningfully improve outcomes in clinical HF settings [44,49,50,51].

### 4.2. Trimethylamine N-Oxide (TMAO): Pro-Inflammatory and Pro-Atherogenic Effects

The metabolic pathway encompassing choline, phosphatidylcholine, L-carnitine, and betaine culminates in the synthesis of trimethylamine (TMA), which is mediated by modified gut microbiota that utilize an array of enzymes most notably, TMA synthase [52]. Subsequently, this TMA undergoes oxidation to generate trimethylamine N-oxide (TMAO) within the hepatic environment, a process facilitated by hepatic flavin monooxygenases (FMO) [53]. Consequently, fluctuations in TMAO concentrations can be correlated with alterations in the gut microbiota composition. In patients afflicted by chronic heart failure, the integrity of the intestinal mucosal barrier is significantly compromised, which results in increased permeability; this phenomenon allows for the unhindered entry of TMAO into the circulatory system, leading to elevated plasma levels. Furthermore, TMAO functions to augment platelet reactivity by modulating stimulus-dependent calcium signaling pathways. Thus, it exacerbates conditions such as atherosclerosis and thrombosis, which are critical to the pathogenesis of heart failure [54]. However, understanding these interactions is essential for developing targeted therapeutic strategies.

Research emphasizes that TMAO serves a crucial function in the regulation of gut microbiota, cholesterol metabolism, and metabolic stress, particularly in scenarios characterized by heightened cholesterol overload [54]. However, the consequences of this correlation are intricate, warranting additional scholarly exploration. Elevated TMAO levels promote the infiltration of macrophages, which are saturated with cholesterol, thereby significantly affecting lipid and hormonal homeostasis. This dynamic interplay ultimately contributes to the pathogenesis of CVDs [55]. Nevertheless, the complexities embedded in these interactions are profound because they encompass a multitude of metabolic pathways. Although the exact mechanisms remain under scrutiny, it is increasingly evident that the presence of TMAO holds extensive ramifications for cardiovascular health.

Engaging the NF-κB pathway, TMAO catalyzes the upregulation of inflammatory genes within aortic endothelial cells and vascular smooth muscle cells [52]. This compound not only amplifies the expression of vascular cell adhesion molecule-1 but also fosters monocyte adherence, thereby activating both NF-κB and protein kinase C. These effects may, however, accelerate the development of chronic heart failure; they exacerbate endothelial dysfunction while concurrently undermining self-repair mechanisms and instigating an inflammatory response. Although TMAO predominantly stimulates NF-κB, it additionally activates the NLRP3 inflammasome, generating a proinflammatory milieu that has been substantiated in both human aortic endothelial cells and carotid artery endothelial cells. This observation implicates TMAO in its role regarding endothelial dysfunction and CVD [56].

**Figure 3:**
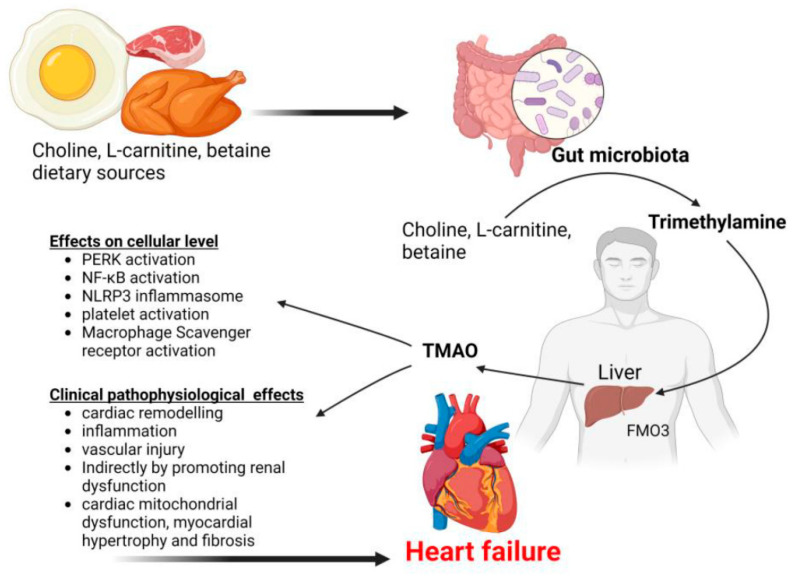
TMAO-mediated heart failure pathogenesis pathways [60].

Moreover, another avenue through which TMAO contributes to HF involves the induction of aortic stiffness, an increase in systolic blood pressure, and the activation of platelets, thereby leading to a hypercoagulable state [57]. TMAO significantly aggravates hypertension due to its direct binding and subsequent activation of protein kinase R-like endoplasmic reticulum kinase (PERK). This activation promotes apoptotic inflammatory responses, which, in concert with the generation of reactive oxygen species, precipitates vascular injury and cardiac remodeling. Thus, it results in elevated blood pressure, as demonstrated by numerous studies [58]. However, TMAO’s function extends beyond mere involvement in the pathogenesis of heart failure Figure 3; it has been associated with the onset of a multitude of cardiovascular, metabolic, and cerebrovascular disorders [59]. Although the underlying mechanisms are complex, the ramifications of TMAO in these processes are, undoubtedly, significantly profound.

Choline, betaine, and L-carnitine derived from dietary sources undergo conversion into trimethylamine through the action of gut microbiota. Subsequently, this trimethylamine is metabolized into TMAO by the enzymatic activity of flavin-containing monooxygenase 3 (FMO3) within the liver [54]. TMAO serves to activate a multitude of intracellular signaling pathways, which in turn facilitate vascular and cardiovascular pathological alterations that can culminate in heart failure. Although the specific mechanisms remain an area of active research, it is evident that key players such as PERK (protein kinase RNA-like endoplasmic reticulum kinase), NF-κB (nuclear factor kappa-light-chain-enhancer of activated B cells), and NLRP3 (NLR family pyrin domain containing 3) are involved. This intricate interplay underscores the complexities of metabolic processes and their implications for cardiovascular health [56]. However, further elucidation of these pathways is necessary to fully appreciate the ramifications of TMAO in pathological states.

### 4.3. Bile Acid

Bile acids fulfill an indispensable function in the intricate composition of bile [42]. In humans, the predominant bile acids, chenodeoxycholic acid, cholic acid (CA) and lithocholic acid are synthesized through the involvement of at least 17 distinct enzymes [61]. These compounds are synthesized from cholesterol via either the classical or neutral pathway, or alternatively, through the acidic pathway [62]. Following their synthesis, they undergo conjugation within the liver and are subsequently secreted into the gut lumen, where they are metabolized by microbiota into secondary bile acids. Bile acids are actively involved in the metabolism of cholesterol, lipids and glucose; they facilitate fat absorption, demonstrating their multifaceted roles [63]. The gut microbiota, under typical physiological conditions, sustains a state of equilibrium, playing an integral role in the development and regulation of the intestinal mucosal barrier. It governs nutrient intake, storage and metabolism, aids in the maturation of immune tissues and helps to prevent the proliferation of pathogenic microorganisms. However, this delicate balance can be disrupted, leading to various health issues. Although essential, the complexity of these interactions necessitates further exploration, because the implications of bile acid metabolism extend far beyond mere digestion.

However, alterations in the bile acid pool can disrupt this balance: resulting in a shift in gut flora distribution that may enable pathogenic microorganisms to flourish. This disruption can lead to pathological conditions, such as inflammatory bowel syndrome, obesity, diabetes, colorectal cancer and cardiovascular diseases (including heart failure, [42]). Although these conditions are varied, they share a common link to changes in gut microbiota; because the health of the gut is intrinsically tied to overall well-being, this relationship merits further investigation.

Conversely, modifications in the composition of microbiota can substantially impact the bile acid pool; thereby, both directly and indirectly, contributing to the pathogenesis of cardiometabolic diseases [64]. Bile acids manifest inotropic, lusitropic and chronotropic effects upon engaging with specific bile acid receptors, such as the muscarinic M2 receptor, Takeda G-protein-coupled receptor 5 (TGR5) and farnesoid X receptor (FXR), all of which are present on cardiomyocytes [63]. These receptors seem to become activated when secondary bile acids are synthesized, a process that is contingent upon the presence of certain gut microbiota species [61]. Although the metabolism of bile acids within the framework of cardiometabolic disease has been extensively reviewed in previous literature [64], this article will not explore that subject in depth; instead, it will underscore the importance of secondary bile acids in the evolution of heart failure. The relationship between bile acid and gut microbiota interactions in the etiology of heart failure is complex, involving multiple pathways. However, it is noteworthy that the majority of studies available in the literature have utilized animal models.

Bile acids generally exert a protective role on cardiac cells; however, certain bile acids may yield detrimental effects. For instance, it is established that hydrophobic bile acids such as lithocholic acid are cytotoxic and have been implicated in the etiology of cardiometabolic diseases because of their pronounced affinity for lipids. Conversely, hydrophilic bile acids, exemplified by ursodeoxycholic acid, demonstrate beneficial impacts on cardiac function by ameliorating myocardial fibrosis [65]. Notably, ursodeoxycholic acid binds to FXR, thereby obstructing nitric oxide synthase inhibitors; this mechanism enhances myofilament function and facilitates myocardial relaxation in cases of HF with preserved ejection fraction. In murine models, the binding of bile acids to TGR5 inhibits NLRP3 inflammasome activation, thus averting inflammatory responses. Moreover, this interaction augments the heart’s capacity to adapt to hemodynamic stress during heart failure through the activation of pro-survival kinases and heat shock proteins [66].

Bile acid receptors, specifically FXR and TGR5, occupy a central position in the realm of heart failure. For example, the stimulation of FXR by secondary bile acids in rodent models has been demonstrated to augment the bile acid ratio while concurrently inhibiting the activation of NF-κB; this action mitigates inflammation and hypertrophic changes within the myocardium [67]. Prolonged NF-κB activation, however, culminates in an upregulation of atrial natriuretic factor expression, thereby contributing to the enlargement of cardiomyocytes [67]. NF-κB functions as an essential transcription factor that enhances the expression of a myriad of genes, including those relevant to inflammation, cellular differentiation, proliferation and apoptosis [68]. In the cytoplasmic milieu of quiescent cells, NF-κB dimers remain in a bound state with inhibitory proteins (IκB), predominantly IκBα and IκBβ [69]. This intricate interplay highlights the multifaceted functions of bile acid receptors in cardiac pathophysiology; although further inquiry is necessary to fully elucidate their mechanisms.

**Figure 4:**
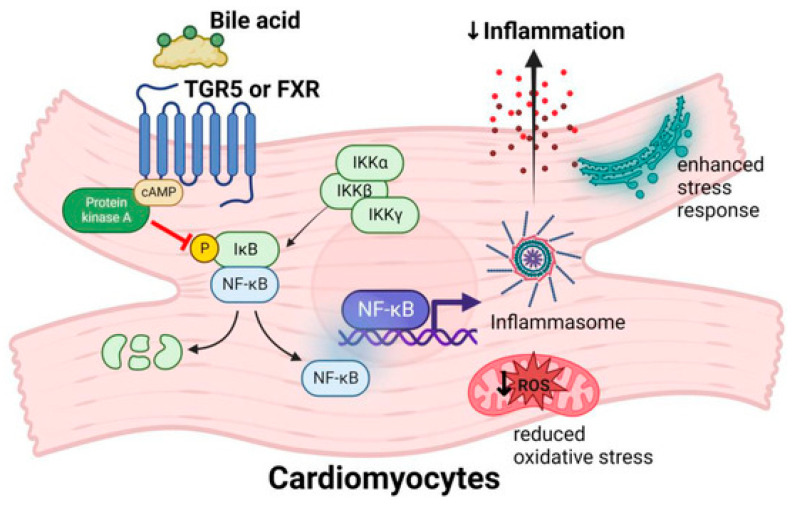
The impact of bile acid signaling on FXR (farnesoid X receptor) and TGR5 (Takeda G-protein-coupled receptor 5) receptors within cardiomyocytes. Bile acids engage these receptors—FXR and TGR5—on cardiomyocytes, thereby activating intracellular signaling pathways that enhance cardiac function. This interaction is significant; however, the precise mechanisms through which these pathways operate remain an area of ongoing investigation. Although the influence of bile acids appears beneficial, further research is essential to fully elucidate their role in cardiac physiology (and potential therapeutic implications) [70].

The activation of NF-κB is contingent upon the phosphorylation of IκB proteins at specific serine residues by the IκB kinase (IKK) a sophisticated protein complex composed of α and β subunits, alongside a regulatory γ subunit. This process of activation, however, results in the degradation of IκB proteins through the 26S proteasome, which is dependent on both ubiquitination and protein kinase activity. As a consequence, NF-κB is liberated, subsequently translocating into the nucleus to initiate the transcription of a diverse array of genes, including those involved in the synthesis of pro-inflammatory cytokines (see Figure 4. Interestingly, the bile acid-mediated activation of TGR5 in murine models has demonstrated an enhancement of cardiac contractility, while simultaneously improving responses to hemodynamic stress. Nevertheless, when the gut microbiota experiences disruption often termed gut dysbiosis, there is a notable decline in essential species that play a crucial role in maintaining adequate bile quantity and homeostasis. This phenomenon includes the activation of the FXR and TGR5 receptors, which ultimately leads to elevated levels of pro-inflammatory cytokines, impaired cardiac function and increased oxidative stress within myocardial cells.

Consequently, the modulation of gut microbiota composition possesses considerable potential to mitigate and avert pathological processes that lead to heart failure. However, additional research is requisite to clarify the specific mechanisms at play particularly because the relationship between gut microbiota and cardiovascular health is intricate and multifaceted.

### 4.4. Phenylacetylglutamine

**Figure 5:**
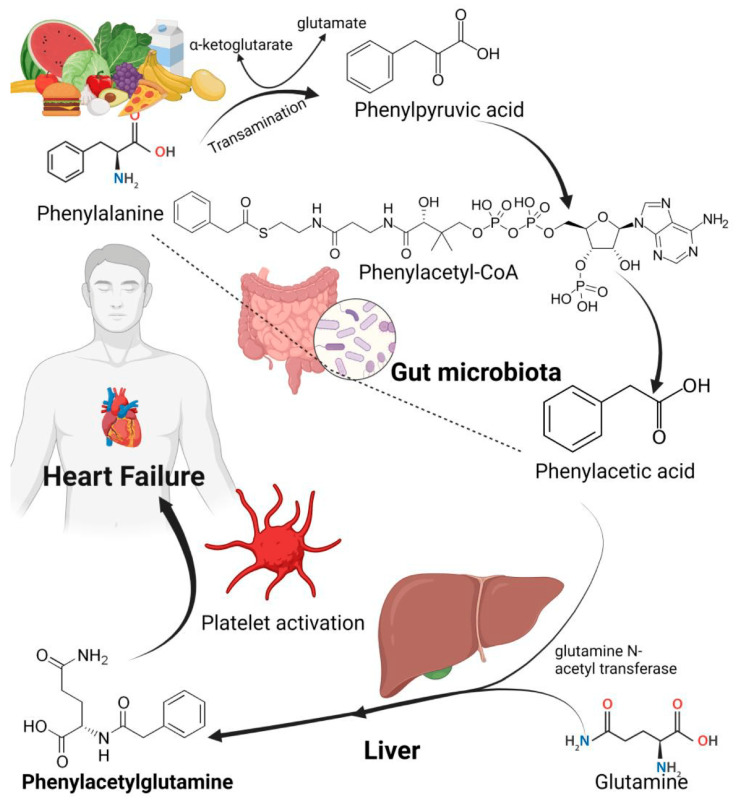
The phenylacetylglutamine formation by the liver enzymes and gut microbiota [76].

Phenylacetylglutamine is intricately linked to the presence and severity of HF [71,72]. This compound, a metabolite produced by the gut microbiota, arises from its nutrient precursor, phenylalanine, an amino acid deemed nutritionally essential because it undergoes conversion to phenylpyruvate within the gastrointestinal tract [73]. Furthermore, the gut microbiota catabolizes phenylacetylglutamine, yielding both phenylpyruvate and phenylacetic acid. Subsequently, in the liver, phenylacetylglutamine is synthesized from phenylacetic acid and glutamine via an amino acid acetylation process, which is catalyzed by the enzymes phenylacetyltransferase and glutamine N-acetyl transferase ([74]; see Figure 5). Notably, these enzymes facilitate the reaction involving the substrates phenylacetyl-CoA and L-glutamine, resulting in the formation of CoA, alpha-N-phenylacetyl-L-glutamine and phenylacetic acid [75]. However, the complexity of these biochemical pathways underscores the significance of phenylacetylglutamine in metabolic processes related to cardiovascular health.

Through its complex interplay with G protein-coupled receptors (GPCRs) and adrenergic receptors (ADRs), phenylacetylglutamine has been shown to significantly affect thrombosis risk by enhancing platelet function; this, in turn, creates a state of hyperresponsiveness among platelets. Such a phenomenon can ultimately lead to the onset of myocardial infarction within the framework of coronary heart disease [73]. However, the engagement of phenylacetylglutamine with GPCRs and ADRs also fosters an overactive sympathetic nervous system, thus exacerbating HF [77]. In a noteworthy recent clinical trial, Romano et al. found that circulating plasma levels of phenylacetylglutamine were not only concentration-dependently associated with HF, indicating that progressively higher circulating levels were linked with greater disease severity metrics particularly reduced ventricular ejection fraction and increased N-terminal pro-B-type natriuretic peptide [72]. This association reflects a graded relationship based on endogenous plasma concentrations rather than an administered dose effect, emphasizing the physiological relevance of phenylacetylglutamine levels in HF progression. Their findings compellingly suggest a clinical and mechanistic connection between HF and the gut microbiota metabolite phenylacetylglutamine.

Research has illuminated the critical significance of phenylacetylglutamine (indeed), which plays a pivotal role in HF; it not only attenuates the contraction of cardiomyocyte sarcomeres but also modifies gene expression related to B-type natriuretic peptide [72]. A recent inquiry, conducted by Fang et al., employed 16S rRNA sequencing techniques to scrutinize patients diagnosed with coronary artery disease (CAD). Their findings revealed a significant correlation between dysbiosis and elevated levels of microbiota-derived phenylacetylglutamine synthesis, particularly in relation to in-stent stenosis and hyperplasia in CAD patients [78]. However, despite the relatively limited volume of research focusing on phenylacetylglutamine, emerging evidence posits that this gut metabolite is associated with a spectrum of CVDs. This correlation accentuates its potential as a target for therapeutic intervention aimed at modulating gut microbiota-derived metabolites to ameliorate CVDs [79]. Although the implications of these findings are substantial, ongoing research remains imperative because a comprehensive understanding of these intricate interactions has yet to be fully realized.

While an increasing amount of research has established strong links between gut dysbiosis, microbial metabolites like TMAO and SCFAs, and HF progression, it is worth noting that much of the evidence comes from observational studies, smaller cohorts, and preclinical models. As such, the mechanisms are largely associative or hypothesis-generating, rather than definitive. High-quality, longitudinal, and randomised control studies are required to demonstrate causation, provide clarity regarding mechanistic pathways, and determine if interventions targeting the microbiome can produce meaningful changes in HF outcomes. Therefore, we present the conclusions of this review, taking into account the limitations and findings of the existing evidence.

In particular contexts pertaining to cardiac insufficiency, elevated concentrations of phenylacetylglutamine function as a critical risk biomarker for the emergence of HF and its ensuing deleterious effects, which may encompass renal dysfunction and mortality [77]. Consequently, phenylacetylglutamine arises as both a prognostic indicator and a risk component within the sphere of heart failure. Moreover, a plethora of investigations [80,81,82,83] have substantiated that phenylacetylglutamine correlates with an increased likelihood of acute ischemic stroke. This heightened risk may present itself in various manifestations: for example, the occurrence of white matter hyperintensities in patients enduring acute ischemic stroke, the intensity of coronary atherosclerosis [82] and the prevalence of coronary artery disease [84]. However, these associations do not remain limited to these particular conditions; there exists a conceivable connection to lethal prostate cancer [83]. Although the fundamental mechanisms are yet to be elucidated, these revelations underscore the necessity of understanding the ramifications of phenylacetylglutamine.

In light of this context, it is essential (imperative, indeed) that subsequent investigations be undertaken to scrutinize these interrelations with heightened rigor. However, the complexity of such analyses cannot be overstated; although preliminary findings may furnish a foundation, they merely scratch the surface. Furthermore, it is crucial to recognize that deeper explorations are necessary, not only to validate initial observations but also to unveil nuanced dynamics that may otherwise remain obscured.

## 5. Clinical Evidence Linking the Microbiome to HF

Emerging research has illuminated the phenomenon of gut dysbiosis among individuals diagnosed with HF, which is characterized by an imbalance in microbial diversity and abundance. Investigations indicate that the gut microbiota composition in HF patients contrasts markedly with that of healthy individuals. For instance, these patients frequently exhibit a diminished diversity in advantageous bacterial taxa, such as Faecalibacterium and Roseburia recognized for their role as producers of SCFAs [43]. Simultaneously, there is a discernible proliferation of pathogenic bacteria, including Escherichia coli and Enterococcus. Notably, a pivotal study conducted by Kummen et al. [85] elucidated a remarkable shift in microbial composition, revealing that HF patients possess a heightened abundance of pro-inflammatory bacterial strains alongside reduced levels of SCFA-generating bacteria. This dysbiosis is posited to be a contributing factor to systemic inflammation, endotoxemia and immune dysregulation; all of which are intricately implicated in the pathophysiology of HF. However, the complexity of these interactions necessitates further exploration to comprehend fully their implications. Although the findings are compelling, the nuances of microbial interactions in HF present challenges, because they reveal a multifaceted landscape where therapeutic interventions may be required to restore microbial balance.

It is imperative to acknowledge that the relationship between gut microbiota and cardiac function is intricate. Although specific bacterial taxa may be reduced, the existence of pathogenic strains prompts further inquiry regarding their potential role in the exacerbation of HF. Consequently, a more sophisticated comprehension of microbial interactions becomes essential for the formulation of targeted therapeutic strategies. Moreover, the compromised functionality of the intestinal barrier observed in individuals with heart failure has been correlated with the presence of gut-derived lipopolysaccharides (LPS); these compounds, however, intensify inflammation and facilitate myocardial dysfunction [86]. Although the nature of this relationship is multifaceted, it is evident that this phenomenon profoundly influences patient outcomes, as it underscores the necessity for focused interventions.

Correlations Between Microbial Composition, Metabolite Levels and HF Severity/Prognosis: The complex interaction between the gut microbiota and HF is primarily mediated through its metabolites such as TMAO, SCFAs and secondary bile acids. Elevated concentrations of TMAO, which arise from the microbial degradation of dietary choline and carnitine, have consistently been linked to adverse cardiovascular outcomes; most notably, HF severity and mortality. Research conducted by [87] elucidated that higher plasma TMAO levels in HF patients were predictive of increased long-term mortality, regardless of traditional risk factors. SCFAs, particularly butyrate, acetate and propionate, are acknowledged for their anti-inflammatory properties and their role in enhancing the integrity of the gut barrier. However, it is essential to recognize that HF patients often exhibit diminished SCFA levels, which correlates with heightened gut permeability and systemic inflammation. Although this relationship necessitates further exploration, it underscores the intricate dynamics between microbial metabolites and cardiovascular health.

The discernible reduction in concentrations of SCFAs has been significantly correlated with the progression of a variety of diseases; this phenomenon is predominantly due to the critical function these metabolites serve in sustaining immune homeostasis and alleviating oxidative stress. Their deficiency may, therefore, intensify clinical outcomes. Furthermore, the altered bile acid profiles observed in individuals suffering from HF unveil a considerable disruption within the gut-liver axis. Secondary bile acids those that emerge from microbial alterations of primary bile acids play a crucial role in both lipid metabolism and the inflammatory response. Aberrant signaling of bile acids has been associated with metabolic dysfunction and adverse outcomes in HF. Collectively, these findings underscore a direct relationship between microbial metabolites and the progression of HF, thereby illuminating the complex interplay between gut microbiota and systemic health. However, because of the multifaceted nature of these interactions, further research is warranted to delineate the underlying mechanisms.

The Impact of Comorbidities on Gastrointestinal Health and HF Outcomes: Comorbidities (which frequently correlate with HF) such as diabetes, obesity and chronic kidney disease, tend to aggravate gut dysbiosis. Consequently, this escalation of dysbiosis results in detrimental effects [78]. For example, diabetes is acknowledged for triggering hyperglycemia-associated modifications in the composition of gut microbiota; however, this phenomenon fosters an increase in pro-inflammatory species while concurrently reducing beneficial bacteria. Such alterations intensify systemic inflammation and endothelial dysfunction, thereby exacerbating the advancement of HF. Although the relationship is complex, the ramifications are significant because they highlight the necessity for comprehensive management strategies in individuals experiencing HF.

Obesity functions as a pervasive comorbidity associated with HF, disrupting the complex diversity of gut microbiota, primarily due to dietary habits characterized by an excess of saturated fats and a significant deficiency in fiber intake. These dietary alterations catalyze the synthesis of TMAO and various deleterious metabolites; consequently, they amplify the risk of HF onset while simultaneously aggravating pre-existing conditions [55]. Moreover, the modifications in gut microbiota, induced by obesity are intricately linked to metabolic endotoxemia; this particular phenomenon plays a crucial role in fostering chronic low-grade inflammation and facilitating cardiac remodeling. Chronic kidney disease frequently coexists with HF, profoundly impacting gut health [74]. Uremic toxins (such as p-cresol sulfate and indoxyl sulfate) are produced by dysbiotic microbiota within the realm of CKD. These substances contribute to vascular calcification, oxidative stress and inflammation, ultimately compromising the prognosis for individuals suffering from heart failure.

The intricate interplay (among these elements) underscores the complexity that is inherent in the management of HF in patients who concurrently grapple with obesity and CKD [88]. This complexity is magnified because of the multifaceted nature of these coexisting conditions; however, it is crucial to address them holistically. Although managing HF in such patients presents numerous challenges, understanding the interdependencies among these factors is essential for effective treatment strategies.

The evidence connecting gut dysbiosis (alongside its metabolites) to HF highlights the complex interplay between the gut microbiota and cardiovascular health. Dysbiosis not only intensifies inflammation and metabolic dysfunction; however, it also affects HF prognosis because of its impact on gut-derived metabolites [78]. Furthermore, comorbidities including diabetes, obesity and CKD serve to amplify these effects. This situation underscores the necessity for personalized therapeutic strategies aimed at modulating the gut microbiota, with the goal of enhancing HF outcomes.

The studies concerning gut microbiota in patients diagnosed with heart HF are encapsulated within Table 2. This compilation offers a comprehensive overview; however, it also highlights the complexity of the interactions involved. Although the research presents various findings, the implications remain nuanced because of the multifaceted nature of microbiota influence on health outcomes.

## 6. Therapeutic Implications and Future Directions

### 6.1. Therapeutic Target

Recently published therapeutic guidelines for HF present an extensive array of recommendations regarding the management of HF and its various subtypes. Both non-pharmacological and pharmacological interventions are proposed, organized according to the new Universal Definition and Classification of Heart Failure. Strong class 1 evidence supports most disease phenotypes, except for pharmacological agents specifically targeting HF with preserved ejection fraction (HFpEF) [99]. However, prior studies indicate that chronic HF patients exhibiting elevated TMAO levels experienced poorer outcomes; moreover, TMAO levels did not respond favorably to guideline-based therapies [30,40,100,101]. Adding TMAO to a model that incorporates B-type natriuretic peptide enhanced prognosis. Thus, further investigation into therapeutic mechanisms aimed at manipulating the gut microbiome could prove beneficial [102]. Although some therapeutic options have been applied to treat other clinical conditions, their application in HF remains largely hypothetical or experimental at this stage, necessitating further validation.

Microbiome-targeted interventions, including diet modification, prebiotics, probiotics, and faecal microbiota transplantation, are emerging adjunctive approaches to HF management. Early experimental studies demonstrate potential benefits in inflammation regulation, metabolic balance, and cardiac remodelling; however, clinical data remain limited, and efficacy in large randomised trials has yet to be confirmed. Future research should prioritise mechanistic studies and standardised human trials to clarify therapeutic relevance and optimise patient selection [38,88,103,104].

Although the randomized controlled trial by Moludi et al. [105] reported reductions in inflammatory markers such as TGF-β and TMAO following probiotic supplementation in AMI patients, the clinical impact of these findings remains uncertain due to the lack of significant improvement in echocardiographic parameters or validated prognostic endpoints. TGF-β and TMAO serve primarily as biological response indicators and are not yet established as independent predictors of HF severity or mortality. This trial therefore highlights a key limitation of the current evidence base: while mechanistic hypotheses are supported by biomarker changes, clinical translation remains limited [104]. Most available trials remain small, heterogeneous, and mechanistically inferential rather than causative, and robust therapeutic benefit in heart failure has not yet been demonstrated. Larger multi-centre RCTs incorporating hard clinical outcomes and microbiome-phenotype stratification are required to determine whether microbiome-modulating therapies can achieve meaningful improvements in HF management [106].

Similarly, preliminary animal and exploratory human studies evaluating interventions such as renal denervation suggest possible microbiome-related effects; however, these remain investigational and should not currently be interpreted as confirmed therapeutic strategies.

### 6.2. Dietary Interventions

The reduction in dietary red meat consumption has been demonstrated to effectively attenuate the intake of TMAO precursors; patients who adhere to a Mediterranean dietary regimen have exhibited a significant decrease in both CVD and mortality risk [42]. Indeed, clinical investigations furnish compelling evidence that a transition to a diet free of red meat can precipitate rapid declines in plasma TMAO levels (as indicated by [107]). Furthermore, diets rich in fiber have shown promise in mitigating the onset of HF during preclinical studies, a phenomenon believed to be linked to an increased production of the SCFA acetate. This, in turn, engenders beneficial cardioprotective effects and strengthens the maintenance of the gut barrier [108]. However, a typical Western dietary pattern, characterized by low fiber and high saturated fat content has been associated with heightened intestinal permeability, thereby elevating circulating lipopolysaccharides (LPS) and contributing to endotoxemia.

Although these dietary modifications have been associated with favorable outcomes concerning cardiac function and HF biomarker levels, the impact of additional simultaneous lifestyle interventions remains ambiguous. This uncertainty continues to exist because, although some studies indicate positive correlations, others produce inconclusive findings. However, the necessity for a thorough understanding is paramount; this complexity warrants further exploration.

The imperative for a comprehensive investigation into the synergistic effects of dietary modifications in conjunction with established lifestyle changes among individuals diagnosed with HF cannot be overstated [42]. Exercise increasingly emerges as a pivotal modulator of the gut microbiota (this), yielding beneficial repercussions for cardiac function, particularly due to its correlation with heightened levels of SCFAs, particularly butyrate [109]. Addressing the phenomenon of sleep fragmentation, which is often encountered in individuals suffering from HF, has also been posited as possessing therapeutic potential; however, a recent preclinical study utilizing murine models revealed that while the isolated induction of sleep fragmentation and HF instigated alterations in the gut microbiome, the concurrent onset of both conditions did not yield any supplementary effects. Therefore, further inquiry is essential for elucidating the intricate interrelations among sleep disturbances, modifications in the gut microbiome and the progression and outcomes of HF [110].

### 6.3. Renal Denervation

Recently, renal denervation (RDN) has emerged as a promising therapeutic intervention for HF, given its capacity to diminish global sympathetic tone; this reduction may effectively address the intricate role that the sympathetic nervous system plays in the pathophysiology and evolution of HF [111]. Previous studies have demonstrated that RDN confers benefits in the management of hypertension, a condition that can significantly contribute to the onset of HF. Moreover, it has recently been established that RDN may serve as a safe and efficacious treatment for HF with preserved ejection fraction, exhibiting advantages that appear to be independent of fluctuations in blood pressure [112]. The interplay between RDN and the gut microbiome is particularly noteworthy; Ref. [113] reported that RDN successfully reversed aberrant alterations in the gut microbiome of rats suffering from chronic HF, characterized by increased populations of beneficial bacteria and a concomitant reduction in detrimental bacterial species. However, to substantiate these preliminary observations, further preclinical and clinical investigations are imperative, as they will facilitate a more nuanced understanding of the underlying mechanisms that drive these notable changes.

### 6.4. Fecal Microbiota Transplantation (FMT)

Fecal Microbiota Transplantation seeks to transfer functional bacteria from healthy individuals to patients, thus modifying the composition of gut microbiota [109]. This procedure has demonstrated effectiveness in treating refractory Clostridioides difficile infections and inflammatory bowel disease. However, the applicability of FMT in HF patients remains ambiguous; because, to our knowledge, no studies have been conducted involving HF patients thus far [16,38,41]. Furthermore, one must take into account the risks associated with infection, endotoxin transfer and rejection [16]. Although the potential benefits seem promising, caution is indeed warranted.

### 6.5. Areas for Future Research

To augment our understanding of the gut microbiome’s role in HF and its associated therapeutic ramifications, a multitude of domains for prospective inquiry warrant thorough scrutiny.

Personalized microbiome-based interventions, which necessitate microbiome profiling for customized therapies, emerge as particularly salient [114]. Individual variability in gut microbiota composition compels the establishment of a personalized framework for microbiome-centric interventions. Future investigations should prioritize the identification of specific microbial signatures that correlate with HF subtypes, thereby facilitating the development of targeted therapeutic strategies. Dietary modifications, furthermore, epitomize another vital area; research is imperative to determine the most advantageous dietary patterns for modulating gut health in patients suffering from HF [114]. This exploration must incorporate the effects of fiber-rich diets, polyphenols, and certain prebiotics on both gut microbiota composition and HF-related outcomes; however, challenges persist in understanding these complex interactions. Although the trajectory of research appears promising, the nuances of microbial interactions must not be overlooked because they play a pivotal role in shaping therapeutic approaches.

Probiotics and postbiotics undoubtedly warrant meticulous examination [115]; the identification of probiotic strains capable of enhancing beneficial metabolites such as SCFAs or mitigating deleterious metabolites like TMAO [56] may yield more effective management strategies for HF. Furthermore, postbiotics metabolites produced by probiotics exhibit substantial promise as direct therapeutic agents within this context. However, the integration of these disparate elements into a cohesive therapeutic framework presents a challenge that merits comprehensive exploration. FMT: Although FMT has demonstrated potential in addressing gastrointestinal disorders, its efficacy and safety concerning patients with HF remain largely uncharted [86]. Consequently, future clinical trials should rigorously evaluate its role in restoring gut homeostasis in the setting of HF. This line of inquiry is crucial because understanding the complex interplay between gut microbiota and heart failure could reveal significant insights into innovative therapeutic avenues.

However, the multifaceted (and often complex) nature of the human microbiome necessitates a sophisticated approach to these explorations. This complexity arises because the interactions within the microbiome can vary significantly, influencing health outcomes in ways that are not yet fully understood. Although there is a growing body of research, the intricacies involved require careful consideration and nuanced methodologies. Consequently, researchers must grapple with various factors that can affect their findings, including environmental influences and genetic predispositions. Thus, understanding the human microbiome is not merely an academic pursuit; it is a vital component of advancing our knowledge in health sciences.

Multi-Omics Approaches: Metagenomics (the exhaustive profiling of the genetic potential inherent within the gut microbiome) offers the potential to illuminate novel microbial genes and pathways that are profoundly intertwined with the pathophysiology of HF [110]. However, advanced metabolomic studies are crucial for delineating the complete spectrum of gut-derived metabolites and their mechanistic affiliations with the progression of HF [80]. This pursuit can dramatically enhance the identification of new biomarkers and therapeutic targets. Transcriptomics and proteomics, conversely, allow for an in-depth examination of gene expression and protein interactions within both the gut microbiome and host tissues. Although these investigations might appear divergent, they furnish more profound insights into the communication between host and microbiota, underscoring its critical role in HF. Integrated multi-omics approaches comprising metagenomics, metabolomics, transcriptomics, and proteomics can produce a comprehensive understanding of the intricate gut-heart axis. Because such integrative methodologies have the capacity to unveil complex interactions, they are indispensable for uncovering novel pathways that could function as targets for therapeutic intervention.

Role of the Gut-Liver-Heart Axis: Given the liver’s essential role in metabolizing products derived from the gut, future investigations must explore the complex interactions among gut microbiota, hepatic functionality, and cardiac well-being [116]. The examination of bile acid metabolism and its resultant effects on HF progression is, however, especially pertinent. Although a substantial amount of information exists, the intricacies of these interrelations necessitate additional inquiry because they could provide valuable perspectives on innovative therapeutic approaches. This investigation may reveal pathways that have been previously neglected, thereby enriching our comprehension of systemic health.

Microbiota and Host Immune Interactions: Further investigations are requisite to elucidate the mechanisms by which gut microbiota modulates systemic immune responses in patients with HF [117]. Understanding these intricate interactions may, however, unveil novel therapeutic strategies aimed at mitigating inflammation and enhancing clinical outcomes. This is particularly important because the relationship between gut microbiota and systemic immunity remains a complex area of study. Although progress has been made, the depth of this connection necessitates more comprehensive research; thus, it is crucial to continue exploring this multifaceted domain.

Gut Barrier Integrity and HF Progression: Exploring the mechanisms that contribute to increased gut permeability in HF and its ensuing repercussions such as endotoxemia holds critical significance. Research ought to evaluate diverse interventions aimed at reinstating gut barrier integrity, not merely to reduce systemic inflammation but also to improve overall health outcomes. However, the intricate nature of these interactions demands a comprehensive understanding of the fundamental biological processes. Although notable advancements have been achieved, continued investigation is imperative because the ramifications of gut health reach far beyond the gastrointestinal system; this highlights the urgent need to tackle these concerns in a holistic manner.

#### Longitudinal Studies and Clinical Trials

Long-Term Studies: The preponderance of modern research primarily depends on cross-sectional data; however, longitudinal studies are essential for illuminating the dynamic changes in gut microbiota as they evolve during the progression and treatment of HF. Clinical Trials: Randomized controlled trials that assess the efficacy of microbiome-based interventions (including prebiotics, probiotics, dietary modifications, and FMT [86]) are vital for substantiating their therapeutic potential. Moreover, the influence of comorbidities on gut-heart interactions warrants thorough investigation. Future studies ought to explore how comorbid conditions such as diabetes, obesity, and chronic kidney disease impact gut dysbiosis [78] and HF outcomes. Understanding these interactions is crucial because it can guide the development of more comprehensive treatment strategies. Microbial-Derived Biomarkers for Diagnosis and Prognosis: The quest for reliable gut-derived biomarkers for HF diagnosis, risk stratification, and monitoring of treatment response represents a promising research trajectory.

Biomarkers such as TMAO, SCFAs, and bile acids necessitate further validation across a variety of patient populations. This emphasis on gut microbiota has the potential to revolutionize our approach to HF; however, challenges persist in the standardization of methods and interpretations. Although promising, this evolving landscape requires careful consideration because it impacts clinical practice significantly.

## 7. Conclusions

Emerging research highlights the complex function of the gut microbiota and its metabolites in HF pathogenesis. Gut dysbiosis, defined by altered microbial diversity and composition, is widespread in heart failure patients, leading to increased gut permeability, systemic inflammation, and unfavorable cardiac remodeling. Significant metabolites such as TMAO, SCFAs, secondary bile acids, and phenylacetylglutamine have been linked to cardiovascular dysfunction, emphasizing the need of studying the gut-heart axis. Diabetes, obesity, and chronic renal disease all worsen gut dysbiosis, impacting the course and prognosis of HF. While therapy aimed at the gut microbiota, such as nutritional interventions, probiotics, prebiotics, and FMT, has shown promise, substantial clinical data supporting their efficacy in HF care are currently sparse. Advances in multi-omics techniques and tailored microbiome-based therapeutics open up new possibilities for future research, allowing for a better knowledge of the gut-heart interplay and paving the way for novel, focused interventions. Multidisciplinary research combining microbiology, bioinformatics, and cardiology is crucial to addressing these issues. To validate treatment approaches and apply microbiome knowledge to clinical practice, longitudinal investigations and randomized controlled trials are essential. By utilizing these discoveries, we may be able to transform the treatment of HF, enhance patient outcomes, and deepen our comprehension of the intricate connection between gut microbiota and cardiovascular health.

## Figures and Tables

**Table 1:** Analytical Methods for Microbiome Research.

Method	Principle	Strengths	Limitations
**Culturomics**	Traditional approach where live microorganisms are cultivated on selective media under controlled conditions.	- Cost-effective - Widely accessible - Allows detailed phenotypic analysis (e.g., pathogenicity, antibiotic resistance, metabolic functions) - Suitable for aerotolerant organisms and rare bacteria poorly represented in databases.	- Labor-intensive - Limited to cultivable microbes - Results influenced by media and conditions - Challenges in studying strict anaerobes or capturing microbe interactions in mixed communities.
**Polymerase Chain Reaction (PCR)**	Sequencing-based method targeting specific microbial *genes* for detection and quantification (e.g., qPCR, RT-PCR).	- Rapid and widely available - Cost-effective - Provides absolute abundance of target taxa - High dynamic range.	- Targets limited number of genes/microbes - Unable to detect unknown taxa - Susceptible to PCR biases - Results depend on primer and probe specificity.
**16S rRNA Gene Sequencing**	Amplification and sequencing of hypervariable regions of the 16S rRNA gene, followed by matching reads to reference databases.	- Commonly used for taxonomic profiling - Identifies cultured and uncultured microbes at the genus level - Relatively rapid and cost-efficient - Well-developed bioinformatic pipelines.	- Provides relative abundance only - Low resolution at species/strain level - Limited to bacteria and archaea; fungi require specialized primers - Cannot distinguish live versus dead microbes - Lacks functional insights.
**Shotgun Metagenomics**	High-throughput sequencing of all DNA fragments in a sample, followed by computational assembly and taxonomic/functional annotation.	- Identifies bacteria, archaea, viruses, and fungi - Provides species and strain-level resolution - Offers functional potential characterization - Requires minimal DNA input (<1 ng).	- Computationally intensive - Costly (though decreasing) - Requires advanced bioinformatics - Sequencing host DNA may confound results - Reproducibility remains uncertain.
**Meta-transcriptomics**	Sequencing of RNA transcripts to analyze microbiome gene expression.	- Enables real-time profiling of microbial activity - Quantitative and high resolution - Integrates functional insights with metagenomics for comprehensive analysis.	- Technically complex and costly - RNA instability poses challenges - Requires paired metagenomic data for optimal interpretation - Limited correlation with meta-proteomic outputs.
**Meta-proteomics**	Mass spectrometry (MS)-based quantification of microbiome-derived proteins and peptides.	- Provides functional insights into microbial protein expression. - Can reveal host-microbe protein interactions.	- Technically challenging - Requires high-quality reference libraries - Limited sensitivity for low-abundance proteins.
**Metabolomics**	Identification and quantification of microbial metabolites using chromatography and MS.	- Rapid and versatile - Suitable for various sample types (e.g., feces, urine, plasma) - Facilitates discovery of both known and novel metabolites - Low sample requirement.	- Targeted metabolomics identifies known compounds but misses unknowns - Untargeted methods are less quantitative and challenging for metabolite annotation - Limited ability to link metabolites to specific microbial taxa.
**Multiomics**	Integration of multiple data types (e.g., genomics, transcriptomics, proteomics, metabolomics) from the same or concurrent samples.	- Offers a comprehensive systems-level view of microbiome-host interactions - Generates novel hypotheses.	- Costly and computationally demanding - Analytic methods and pipelines are still being standardized.

**Table 2:** Studies on gut microbiota composition in patients with HF.

Reference	Patient Type	Age (Years)	Sample Size	Method	Key Findings
[89]	Acute HF or exacerbation of chronic HF	47.4 ± 2.8 (younger HF) 73.8 ± 2.8 (older HF)	HF < 60: *n* = 12 HF > 60: *n* = 10 Controls: *n* = 12	16S rRNA	↓ *Eubacterium rectale*, *Dorea longicatena* Depletion of *Faecalibacterium* in older patients
[90]	Chronic HF	67 ± 2	Chronic HF: *n* = 22 Controls: *n* = 22	Fluorescence in situ hybridization	↑ *Eubacterium rectale*, *Faecalibacterium*
[91]	Chronic HF	65 ± 1.2	HF: *n*= 60 Controls: *n* = 20	Traditional culture techniques	↑ *Campylobacter*, *Shigella*, *Salmonella*, *Yersinia enterolytica*, *Candida*
[92]	Chronic HF	60.69	HF: *n* = 29 Controls: *n* = 30	16S rRNA	↓ *Ruminococcaceae*, *Lachnospiraceae*, *Dialister* ↑ *Enterococcus*, *Enterococcaceae*
[92]	Chronic HF (NYHA III-IV)	65–86	NYHA III HF: *n* = 29 NYHA IV HF: *n* = 29 Controls: *n* = 22	16S rRNA	NYHA III: ↑ *Escherichia*, *Bifidobacterium* NYHA IV: ↑ *Klebsiella*, *Lactobacillus*
[93]	Chronic HF (70% exacerbation, 30% stable)	65 ± 3.2	HF: *n* = 20 Controls: *n* = 20	16S rRNA	↓ *Coriobacteriaceae*, *Erysipelotrichaceae*, *Ruminococcaceae* (family level) ↓ *Blautia* (genus level)
[85]	Chronic HF	NA	Discovery: *n* = 40 Validation: *n* = 44 Controls: *n* = 266	16S rRNA	↓ *Lachnospiraceae* family
[94]	Stable chronic HF (ischemic/dilated cardiomyopathy)	58.1 ± 13.3	HF: *n* = 53 Controls: *n* = 41	16S rRNA	↑ *Ruminococcus gnavus* ↓ *Faecalibacterium prausnitzii*
[95]	HF with preserved ejection fraction (HFpEF)	40–70	HFpEF: *n* = 26 Controls: *n* = 67	16S rRNA	↓ *Ruminococcus* spp.
[96]	Chronic HF	65 ± 3.17	HF: *n* = 26 Controls: *n* = 26	16S rRNA	↑ *Escherichia*, *Shigella*, *Ruminococcaceae*, *Lactobacillus*, *Atopobium*, *Romboutsia*, *Streptococcus*, *Haemophilus*, *Klebsiella*
[97]	Non-ischemic HF with reduced ejection fraction (HFrEF)	18–70	HFrEF: *n* = 28 Controls: *n* = 19	16S rRNA	↑ *Streptococcus* spp., *Veillonella* spp. ↓ *SMB53*
[98]	Acute decompensated HF/acute worsening of chronic HF	72 ± 18	HF: *n* = 22 Controls: *n* = 11	16S rRNA	↑ *Actinomycetota* (phylum), *Bifidobacterium* (genus) ↓ *Megamonas* (genus)

## Data Availability

No new data were created or analyzed in this study.

## Abbreviations

HF	Heart Failure
SCFAs	Short-Chain Fatty Acids
TMAO	Trimethylamine-N-oxide
LPS	Lipopolysaccharides
BAs	Bile Acids
HIF	Hypoxia-Inducible Factor
FMT	Fecal Microbiota Transplantation
GPR	G-Protein-Coupled Receptors
GDP	Gross Domestic Product
NF-κB	Nuclear Factor Kappa B
NO	Nitric Oxide
NHANES	National Health and Nutrition Examination Survey
CKD	Chronic Kidney Disease
CVDs	cardiovascular diseases
ICIs	Immune Checkpoint Inhibitors
RCT	Randomized Controlled Trial
HRQL	Health-related quality of life
FXR	Farnesoid X Receptor
TGR5	Takeda G-Protein-Coupled Receptor 5
GPCRs	G-Protein-Coupled Receptors
ADRs	Adrenergic Receptors
PERK	Protein Kinase RNA-Like Endoplasmic Reticulum Kinase
QALYs	Quality-Adjusted Life Years
